# Age-related differences of vastus lateralis muscle morphology, contractile properties, upper body grip strength and lower extremity functional capability in healthy adults aged 18 to 70 years

**DOI:** 10.1186/s12877-022-03183-4

**Published:** 2022-06-29

**Authors:** Isobel Jacob, Mark I. Johnson, Gareth Jones, Ashley Jones, Peter Francis

**Affiliations:** 1 Musculoskeletal Health Research Group, Leeds Beckett University, Leeds, England; 2 Centre for Pain Research, Leeds Beckett University, Leeds, England; 3 Department of Science and Health, Institute of Technology Carlow, Carlow, Ireland

**Keywords:** Muscle health, Muscle thickness, Muscle architecture, Function, Strength, Age

## Abstract

**Background:**

There is a lack of of cross-sectional research that has investigated muscle morphology, function, and functional capability in all age-bands of healthy adults. The primary aim of this study was to evaluate age-related differences in indices of vastus lateralis (VL) muscle morphology, function and functional capability in a sample of healthy males and females aged 18-70yrs. Secondary aims were to evaluate relationships between age and VL muscle morphology and function and functional capability.

**Methods:**

B mode Ultrasonography and Tensiomyography were used to measure VL muscle thickness, pennation angle, fascicle length, and contractile properties in 274 healthy adults aged 18-70yrs. Measurements of grip strength and functional capability (1-min chair rise test) were also taken. Data analysis included descriptive statistics, correlations, one-way ANOVAs, and multiple regressions.

**Results:**

Negative correlations were found between age and muscle thickness (r_s_ = -.56), pennation angle (r_s_ = -.50), fascicle length (r_s_ = -.30), maximal displacement (r_s_ = -.24), grip strength (r_s_ = -.27) and the 1-min chair rise test (r_s_ = -.32). Positive correlations were observed between age and the echo intensity of the muscle (r_s_ = .40) and total contraction time (r_s_ = .20). Differences in the indices of muscle health were noticeable between the 18–29 age band and the 50–59 and 60–70 age bands (*p* < 0.05). The interaction of age and level of physical activity predicted changes in the variables (r^2^ = .04—.32).

**Conclusion:**

Age-related differences in muscle health are noticeable at 50 years of age, and age-related differences are larger in females compared to males. It was suggested that the thickness of the VL changed the most with age across the adult lifespan and that physical activity likely acts to abate detrimental change.

**Supplementary Information:**

The online version contains supplementary material available at 10.1186/s12877-022-03183-4.

## Background

Declines in muscle quantity [1–3] and muscle strength [2, 4–6] are associated with declines in functional capability. Functional capability is a term referring to the ability of an individual to perform physical tasks that are important for daily living such as rising from a chair. The strength of a muscle is underpinned not only by muscle size but by muscle morphology, namely pennation angle (PA) [7–9]. Within this project, muscle morphology is a term regarding the structure of a muscle, of which some of the key parameters include PA, fascicle length (FL), tissue composition such as intramuscular fat, connective tissue surrounding the muscle and within the muscle such as aponeuroses. The European Working Group on Sarcopenia in Older People (EWGSOP) [10] recommends classification of older adult muscle health according to differences observed from a young adult reference range. This has contributed to a paucity of data in middle-aged adults. Evidence suggests that alterations in muscle morphology, function and functional capability do not occur simultaneously at different stages of adulthood [11]; this is because research studies have provided evidence that there are larger age-related declines in muscle strength, which may precede changes in muscle mass in older adults [12, 13]. However, due to the lack of data on muscle morphology, function and functional capability in healthy adults aged 18–70 years (yrs) of age, the specific trajectory of age-related differences in aspects of muscle health across 18-70yrs still remains to be investigated.

Studies evaluating the relationship between age and indices of morphology, function and functional capability should control for factors which may have an impact on variables of muscle health. Factors such as the presence of disease or injury may reduce mobility and / or lead to a decline in the external loading on the muscles [14], resulting in atrophy (wasting) of the muscle and possibly accelerating the rate of age-related declines in muscle health sarcopenia [15]. Thus, for the maximal effort tests to detect age-related differences, adults should be healthy, free from disease or injury and able to walk unaided. It could also be argued that indices of muscle health should be evaluated in samples of healthy adults to determine the optimum reference values of muscle morphology, function and functional capability. This may enable adults to evaluate the status of their muscle health in comparison to adults of comparable age who are at the upper echelons of their muscle health.

Muscle thickness (MT), PA and FL play an important role in muscle function [16, 17] and age-related changes in the architecture of a muscle results in alterations to the force producing output of a muscle [17]. Measurements of muscle function have previously included muscle contractile properties [18, 19] and muscle strength. Upper body strength can be measured using hand-held dynamometry which has clinical utility advantages such as portability. Furthermore, normative reference values are available for various populations and therefore, comparisons can be made. Measures of grip strength are correlated to upper leg strength, thus it can be suggested that differences in grip strength may reflect differences in upper leg strength [20]. Investigating factors that underpin muscle function may reveal age-related differences in muscle function that precede differences in muscle strength and functional capability. Hence, measuring MT, PA, FL, contractile properties, strength and functional capability and evaluating the findings may reveal the age-related differences that may be present in adults aged 18-70yrs.

The primary aim of this study was to evaluate age-related differences in indices of VL muscle morphology, function and functional capability in a sample of healthy males and females aged 18-70yrs. Secondary aims were to evaluate relationships between age and VL muscle morphology and function and functional capability.

The specific objectives of this study were to:

1. Assess at which age band, if there are any, differences in MT, PA, FL, muscle quality, contractile properties, upper body strength and lower extremity functional capability become noticeable from the youngest age band (18-29yrs). Directional hypothesis: Age-related differences in the listed parameters of muscle health will be statistically significantly different between those aged 18-29yrs and 50-59yrs and 60-69yrs.
2. Assess whether age is associated with MT, PA, FL, muscle quality, contractile properties, upper body strength and lower extremity functional capability and whether age accounts for the same amount of variance in these variables. Directional hypothesis: The strength of the associations between age and each of variables will not be the same and age will not account for the same amount of variance in each of the variables.
3. Assess the interaction between age and physical activity to explain the variance in MT, PA, FL, muscle quality, contractile properties, upper body strength and lower extremity functional capability. Directional hypothesis: Age and physical activity will account for a larger variance (as reflected by the r^2^ values) in the variables of muscle health compared to age or physical activity alone.

## Methods

### Design

This was a cross sectional study in which measurements of MT, PA, FL, tissue composition (in relation to intramuscular fat), contractile properties, upper limb strength and lower extremity functional capability were taken at one time point for each participant.

The study was conducted in accordance with the Declaration of Helsinki. This study was approved by the Research Ethics Committee of Leeds Beckett University (ethics application number = 50,844). All participants provided written informed consent.

### Participants, recruitment and enrolment

A convenience sample size of 250 healthy, unpaid, female and male adult volunteers aged 18-70yrs was the target sample size. An a priori sample calculation was not performed due to the lack of existing data on measurements of contractile properties and lower extremity functional capability using the 1-min chair rise test in healthy adults aged 18-70yrs. Our priority was to obtain an equal number of participants per age band which then facilitated meaningful comparisons to be made between the age groups without the limitation of unequal numbers in each group which could have affected the findings and conclusions drawn. Participants were recruited via posters advertised around Leeds Beckett University, local sports clubs and Pilates classes. Emails were sent to local neighbourhood network schemes, local community clubs (University of Third Ages) and members of staff at the University. Word of mouth was also used.

**Fig. 1:**
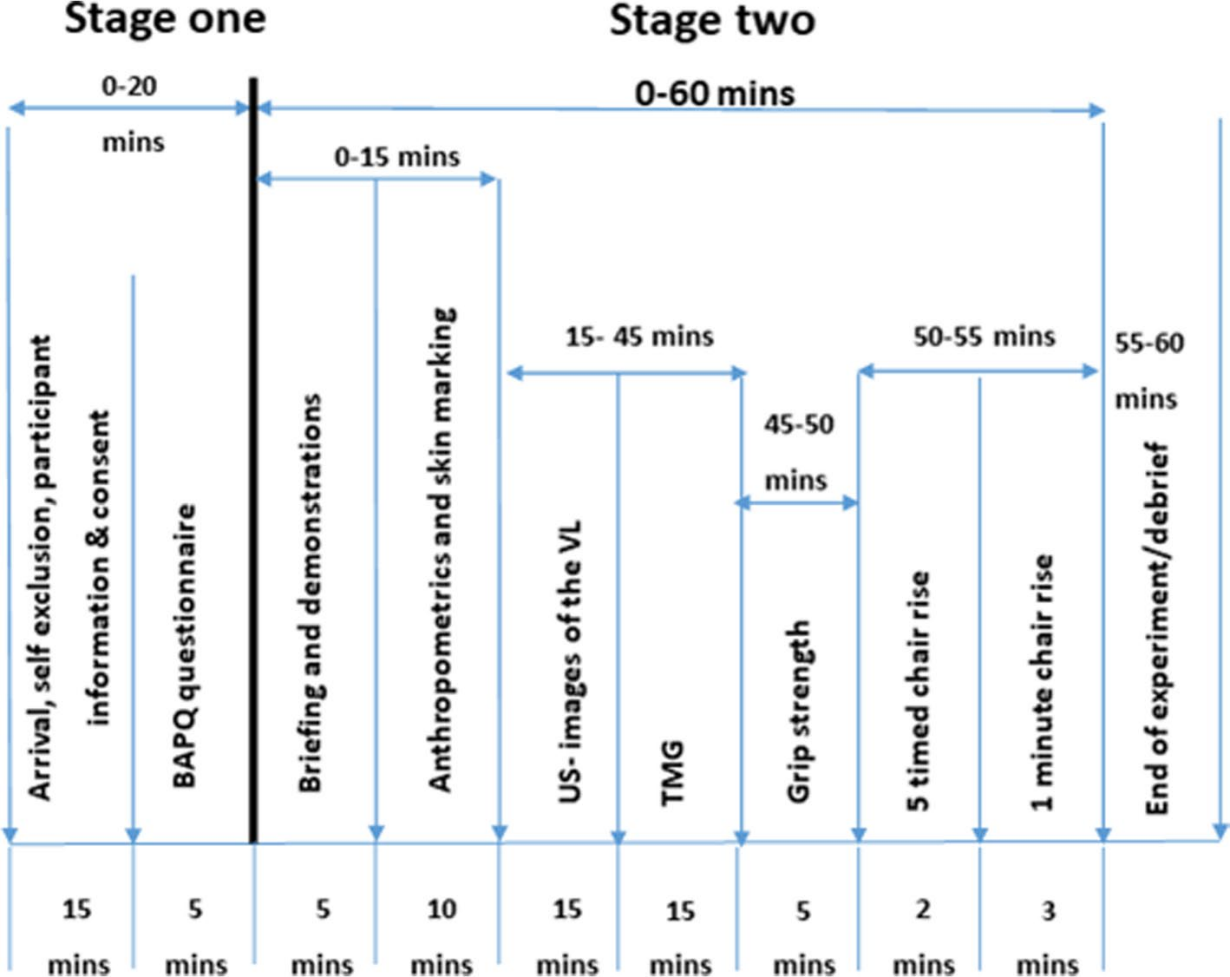
An illustration of the sequence of events undertook for each participant

Participants who were defined as ‘healthy’, independent living and fully mobile were included. Participants were deemed as ‘healthy’ based on the criteria outlined by Greig et al. [21], with allowances to the list of those who: were on blood pressure tablets and their blood pressure taken on the day was within normal limits (120/80); had mild anxiety or depression, including those taking medication; or had controlled asthma. There was no restriction on gender, ethnicity, height or weight. Volunteers who expressed an interest were sent the participant information sheet and the self-exclusion criteria. Those who deemed themselves eligible were invited to the site of testing and were formally screened for eligibility by the primary researcher (IJ) using a pre-screening questionnaire. Volunteers with high blood pressure (over 140/90 mmHg) were excluded from the study. Those who were deemed eligible to take part completed the consent form. All participants were instructed to maintain a normal diet but refrain from consuming caffeine 12 h prior to the study. Participants were also asked to refrain from exercising before taking part in the study. The sequence of events, including timings, for each participant can be seen in Fig. 1.

## Measurements

For specific operational detail please see the Supplementary file S1.

### Anthropometrics

Measurements of height (cm) and weight (kg) were recorded. Each participant’s dominant leg and arm were noted. The femur length (cm) was measured as the distance between the greater trochanter and the lateral femoral condyle, and the girth (cm) of the thigh was measured at the mid-point of the thigh in the transverse plane.

### Physical activity levels

Participants were asked to fill out the International Physical Activity Questionnaire –short form (IPAQ). The IPAQ is comprised of questions based around how physically active the participant has been in the last 7 days. The data from these questions was used to calculate the weighted MET/week for each participant and to classify the physical activity levels of the individuals as either high, moderate or low [22]. The Bone Physical Activity Questionnaire (BPAQ) was used to identify the types and frequency of exercise each participant performed. This was useful when making comparisons between the activity levels and types of exercise performed between the age groups.

### Marking of the vastus lateralis muscle belly

The VL of the dominant leg was marked using a skin-friendly pen. Only the dominant VL was imaged and measured because measurements of upper body strength were taken in the dominant arm, therefore, to enable comparisons between the upper and lower limbs it was decided to only measure the dominant limbs. A mark was made at 50% of the distance between the greater trochanter and the lateral femoral condyle. Keeping in line with the original 50% mark, the medial and lateral borders of the VL muscle were marked by the investigator. The midpoint between these markings was measured and marked X with a skin-friendly pen; this was the site for the probe placement (both Ultrasonography and Tensiomyography (TMG)). The electrode placements were determined by measuring and marking 2.5 cm proximally and distally of the X.

### Muscle thickness, pennation angle, fascicle length and echo intensity

Images of the dominant VL muscle were taken using B mode ultrasonography (LOGIQ e, GE Healthcare, Buckinghamshire*)* with a 5 cm linear array probe to allow for the measurement of MT, PA and FL and echo intensity. The depth of the images was altered to enable a clear image whereby the superficial and deep aponeuroses and 3 clear fascicles could be seen. The frequency was kept constant for every participant to allow measurements of echo intensity to be taken. Participants were supine with the knee extended and relaxed. The investigator followed a measurement protocol [23] which was followed step by step.

The images taken using the B mode Ultrasonography machine were downloaded to imaging software (Image J, v.1.51 k; National Institute of Health; Bethesda; USA), where one measurement of MT, PA, FL and echo intensity was taken. MT was determined as the perpendicular distance between the superficial and deep aponeurosis; PA was determined as the angle at which the fascicle inserted into the deep aponeurosis, and FL was determined as the length of the fascicular path from the superficial and deep aponeuroses. FL was measured using the extrapolation method [24] as the equipment does not have an extended field of view function. Measurements of echo intensity using grayscale analysis was used to reflect the quality of the muscle.

Prior to this study, a reliability study was conducted to assess the reliability of the principal investigator at taking measurements of MT, PA and FL. Two measurements of MT, PA and FL were taken and the intra class correlation coefficient (ICC) and associated 95% confidence intervals (CI) were calculated using a 2-way mixed-effects model. To summarise, excellent levels of intra-rater reliability were achieved for measurements of MT (ICC = 0.95, 95% CI = 0.9 – 098, *p* < 0.01), good levels of intra-rater reliability were achieved for measurements of PA (ICC = 0.88, 95% CI = 0.75- 0.94, *p* < 0.01) and moderate levels of intra-rater reliability were achieved for FL measurements **(**ICC = 0.83, 95% CI = 0.65–0.92, *p* < 0.01). Further details including the standard error of measurement and Bland Altman plots can be found in the study by Jacob et al. [25].

### Contractile properties

Involuntary muscle contractile properties of the dominant VL were measured using TMG. Maximal displacement (Dm) measures the deformation of the muscle belly (mm) during an involuntary muscle contraction, a reduced Dm is suggested to represent an increase in muscle stiffness. Total contraction speed (Tc) reflects the speed of contraction during an involuntary muscle contraction and is measured as the time on the ascending curve between 10 and 90% of Dm [26].

Measurements were taken with the participants supine with the knees extended. Participants were asked to remain relaxed during measurements. Familiarisation measurements were conducted on the non-dominant VL. The skin area was cleaned with alcohol wipes to improve impedance. After the familiarisation, the two self-adhesive electrodes (5 cm x 5 cm) were placed on the marked sites of the dominant leg in preparation for the actual test, with the positive electrode placed on the proximal marking of the muscle and the negative electrode placed on the distal marking of the muscle. The electrodes were attached to the electrical stimulation unit (TMG-S1 doo, Ljubljana, Slovenia). The spring-loaded digital transducer probe (Digital-optical comparator, RLS Ltd, Slovenia) was placed at 50% of the marked belly of the VL as this is the thickest portion of the muscle. The probe and electrodes were kept in the same place for the duration of the testing.

Measurements were taken in a sequential and progressive order until maximal Dm was reached, starting at 30 mA and increasing by 5 mA every 10 s. The 10 s rest period was in line with the rest period reported by Hunter et al. [27]. On stimulation, a monophasic 1 ms pulse stimuli was delivered to the muscle. After each stimulus, the investigator checked the curve on the graph shown on the laptop. The test was terminated if there was no further increase in maximal muscle displacement or if the maximum amplitude that the electrical stimulator was reached (100 mA).

### Strength

Isometric hand grip strength (GS) was measured using a portable Jamar Plus digital handheld dynamometer. All measurements were taken in the dominant hand only. For this study, participants were asked to stand with their arms close to their side and the elbow of the dominant hand bent at 90 degrees when squeezing similar to protocols described by [28]. Participants were given two warm up trials before performing three maximal effort trials. The average of the 3 trials was calculated and recorded as the grip strength measure for each participant.

### Functional capability

Two chair rise tests were conducted to measure functional capability: a 5 timed chair rise test and a 1-min chair rise test. The test was administered using a chair without arms with a height of 45 cm from the ground, which was positioned against a wall to prevent the chair from moving. Participants were instructed to start seated in the middle of the chair, with their back straight, feet shoulder width apart and arms crossed against their chest. The tests started with the participant seated and for the first test, the participants were asked to rise out of the chair as quickly as they could 5 times. The time was recorded, and participants were given a minute’s rest. For the 1-min chair rise, the participants were asked to perform as many chair rises as they could in the time frame. The score was the total number of chair rises executed correctly in the minute.

### Data analysis

Descriptive statistics were calculated for participant characteristics and each of the dependent variables, the data is presented as the mean ± standard deviation and the minimum and maximum range has also been included. Normality of the data was assessed for each age category using a Shapiro–Wilk test. A Welch ANOVA test and Games-Howell post hoc was conducted on the age bands and MT, PA, FL, echo intensity, Dm, Tc, GS and lower extremity functional capability to identify if there were any differences in the dependent variables between the age bands and, if so, at which age band the age-related differences were noticeable from the youngest age band (18-29yrs). Statistical significance was defined as *p* < 0.05. Spearman’s Rank correlation were conducted between age and all of the variables aforementioned in order to determine if age was associate with the variables and the direction of the relationship. Correlation coefficients of + 1/-1 indicated a perfect correlation and correlation coefficients nearer to zero indicated weak correlations. The strength of the correlations was classified according to those defined by Cohen [29]: small = 0.10, moderate = 0.30, large = 0.50. Regression analysis was conducted to determine if the relationships between age and the previously mentioned variables were linear or curvilinear. Polynomial regression analysis was used to confirm curvilinear relationships in which age^2^ significantly contributed (*p* < 0.05) to the regression model for these variables. Multiple regression analysis was conducted to assess the interaction between age and physical activity as a model to explain the variance in each of variables. To determine whether the age-related differences, associations and linearity of the relationships (if there were any) were similar in the females and males, the analysis described above was conducted on the whole sample and by gender.

Statistical analyses were conducted using IBM SPSS statistical package for Windows (version 25).

## Results

### Characteristics of study sample

Two hundred and seventy-four healthy adults aged 18–70 years completed the study (mean ± SD: age = 41.9yrs ± 16.1; height = 169.3 cm ± 10.2; weight = 72.4 kg ± 15.2), the characteristics can be seen in Table 1. Out of the 274 adults 156 females (mean ± SD: age = 43.1yrs ± 16.7; height = 163.9 cm ± 6.83; weight = 65.4 kg ± 10.4) and 118 males took part in the study (mean ± SD: age = 40.5yrs ± 15.5; height = 176.5 cm ± 9.42; weight = 81.6 kg ± 15.7). Comparisons between the youngest (18-29yrs) and oldest (60-70yrs) adults revealed the 60-70 yr old adults had lower measurements of MT, FL, PA and GS (*p* < 0.05) compared to the 18-29 yr olds, see Table 2. The time taken to complete the 5 timed chair rise was longer in the oldest (60-70yrs) adults compared to the youngest (18-29yrs) adults and the older completed a fewer number of chair rises in a minute compared to the youngest (*p* < 0.05). The mean pixels, an indicator of muscle quality, was larger in the older adults compared to the youngest adults (*p* < 0.05). See Supplementary file S2 for the full t test results.

**Table 1:** Participant characteristics for the whole sample (*n* = 274), females (*n* = 156) and males (*n* = 118)

	**18–29**	**30–39**	**40–49**	**50–59**	**60–70**
*Total sample size*	*73*	*50*	*50*	*50*	*51*
*Female sample*	*41*	*26*	*20*	*34*	*35*
*Male sample*	*32*	*24*	*30*	*16*	*16*
**Age**	21.1 ± 1.8 19 – 28	34.4 ± 3.0 30 – 39	44.4 ± 3.4 40 – 49	55.0 ± 2.3 50 – 59	64.1 ± 3.3 60 – 70
**Female**	20.6 ± 1.2 19 – 26	34.8 ± 3.0 30 – 39	44.7 ± 3.5 40 – 49	54.6 ± 2.4 50 – 58	63.8 ± 3.1 60 – 70
**Male**	21.8 ± 2.2 19 – 28	33.9 ± 2.9 30 – 39	44.2 ± 3.3 40 – 49	56.0 ± 1.8 50 – 59	64.9 ± 3.8 60 – 70
**Height (cm)**	170.8 ± 9.4 151.8 – 190.0	172.1 ± 9.5 156.5 – 195.0	171.8 ± 10.2 147.0 – 191.0	166.4 ± 9.0 144.0 – 186.0	164.9 ± 11.2 117.5 – 189.5
**Female**	166.1 ± 7.9 151.8 – 186.5	165.3 ± 5.1 156.5 – 175.5	163.3 ± 6.3 147.0 – 177.5	162.7 ± 7.5 144.0 – 176.0	161.6 ± 5.3 145.5 – 171.5
**Male**	176.7 ± 7.6 147.0 – 191.0	180.1 ± 6.8 170 – 195.0	177.0 ± 8.5 159.0 – 191.0	174.2 ± 6.7 165.0 – 186.0	172.1 ± 16.4 117.5 – 189.5
**Weight (kg)**	68.8 ± 10.3 49.6 – 90.0	75.4 ± 14.0 48.0 – 108.0	78.6 ± 16.3 45.2 – 121.1	68.8 ± 13.0 46.0 – 100.0	70.3 ± 11.5 48.0 – 107.7
**Female**	64.9 ± 8.6 49.6 – 90.0	66.3 ± 10.1 48.0 – 84.4	67.8 ± 12.2 45.2 – 86.5	63.1 ± 10.1 46.0 – 100.0	66.5 ± 11.2 48.0 – 107.7
**Male**	73.7 ± 10.2 52.0 – 89.6	86.0 ± 9.7 64.6 – 108.0	85.3 ± 15.0 63.5 – 121.1	81.1 ± 9.5 62.5 – 93.1	78.4 ± 7.6 68.6 – 92.0
**Femur Length (cm)**	40.7 ± 2.9 34.0 – 47.0	41.0 ± 2.5 36.0 – 46.0	40.9 ± 2.6 36.0 – 46.0	39.5 ± 2.6 35.0 – 46.0	39.7 ± 4.3 34.0 – 63.0
**Female**	40.4 ± 2.9 34.0 – 46.0	39.8 ± 2.0 36.0 – 43.0	38.8 ± 1.6 36.0 – 42.0	38.6 ± 2.1 35.0 – 43.0	38.8 ± 4.7 34.0 – 63.0
**Male**	41.1 ± 3.0 35.0 – 47.0	42.5 ± 2.2 38.0 – 46.0	42.1 ± 2.4 36.0 – 46.0	41.5 ± 2.8 36.0 – 46.0	41.6 ± 2.5 34.0 – 45.0
**Girth (cm)**	53.4 ± 3.8 43.0 – 65.0	54.1 ± 4.6 43.0 – 62.0	53.9 ± 5.7 43.0 – 75.0	50.5 ± 4.3 40.0 – 62.0	50.8 ± 4.2 40.0 – 65.0
**Female**	53.1 ± 4.5 43.0 – 65.0	56.1 ± 3.8 46.0 – 62.0	55.2 ± 55.2 43.0 – 75.0	52.0 ± 4.0 40.0 – 55.5	51.5 ± 3.0 45.0 – 56.0
**Male**	53.1 ± 4.5 43.0 – 65.0	56.1 ± 3.8 46.0 – 62.0	55.2 ± 55.2 43.0 – 75.0	52.0 ± 4.0 40.0 – 55.5	51.5 ± 3.0 45.0 – 56.0

Values are presented as the mean ± standard deviation. The minimum and maximum values are presented underneath the mean ± standard deviation. The values for the whole sample are presented in the first row for each of the characteristics

**Table 2:** Measurements of muscle morphology, muscle function (contractile properties and strength) and lower extremity functional capability from 18 –70yrs of age in the whole sample (*n* = 274), females (*n* = 156) and males (*n* = 118)

	**18–29**	**30–39**	**40–49**	**50–59**	**60–70**
*Total sample size*	*73*	*50*	*50*	*50*	*51*
*Female sample*	*41*	*26*	*20*	*34*	*35*
*Male sample*	*32*	*24*	*30*	*16*	*16*
**Muscle thickness (cm)**	2.0 ± .34 ^*^ 1.44 – 3.24	1.82 ± .37 1.10 – 2.91	1.89 ± .31 1.23 – 2.53	1.56 ± .34^*^ .84 – 2.36	1.42 ± .28^*^ 1.00 – 2.10
Female	1.93 ± .25^*^ 1.44 – 2.66	1.60 ± .20 1.10 – 2.00	1.61 ± .21 1.23 – 1.97	1.41 ± .25^*^ .84 – 1.93	1.33 ± .22^*^ 1.00 – 2.02
Male	2.19 ± .39^*^ 1.65 – 3.24	2.09 ± .36 1.58 – 2.91	2.06 ± .24 1.68 – 2.53	1.89 ± .27^*^ 1.46 – 2.36	1.61 ± .30^*^ 1.08 – 2.10
**Pennation angle (°)**	15.28 ± 2.18^*^ 10.71 – 24.22	14.15 ± 1.88 9.70 – 18.14	14.18 ± 1.81 10.72 – 19.55	12.81 ± 1.65^*^ 8.71 – 16.36	12.23 ± 1.79^*^ 8.77 -18.32
Female	14.94 ± 1.71^*^ 10.71 – 20.66	13.29 ± 1.72 9.70 – 17.99	13.09 ± 1.30 10.72 – 15.80	12.19 ± 1.46^*^ 8.71 – 14.85	11.62 ± 1.41^*^ 8.77 – 15.41
Male	15.71 ± 2.64^*^ 11.94 – 24.22	15.16 ± 1.53 12.62 – 18.14	14.84 ± 1.77 12.13 – 19.55	14.16 ± 1.17 12.15 – 16.36	13.54 ± 1.85^*^ 11.07 – 18.32
**Fascicle length (cm)**	8.34 ± 1.03^*^ 6.21 – 11.82	7.99 ± 1.17 5.69 – 10.55	8.01 ± .75 6.35 – 9.70	7.72 ± .91^*^ 5.92 – 9.90	7.33 ± .89^*^ 6.03 – 9.58
Female	8.24 ± 1.09^*^ 6.21 – 11.82	7.57 ± .92 5.69 – 9.42	7.79 ± .62 6.35 – 8.90	7.54 ± .88^*^ 5.92 – 9.74	7.31 ± .86^*^ 6.03 – 9.21
Male	8.47 ± .94^*^ 6.35 – 10.24	8.48 ± 1.26 6.08 – 10.55	8.15 ± .80 6.75 – 9.70	8.12 ± .86 6.93 – 9.90	7.39 ± .96^*^ 6.22 – 9.58
**Echo intensity (mean pixels)**	56.89 ± 21.28^*^ 18.71 – 99.68	73.20 ± 20.25 33.01 – 112.72	73.14 ± 19.32 24.52 – 104.78	87.61 ± 24.23^*^ 45.81 – 145.34	85.48 ± 29.63^*^ 25.66 – 146.51
Female	62.54 ± 21.11^*^ 29.34 – 99.68	84.46 ± 15.80 49.86 – 112.72	89.13 ± 12.07 52.96 – 104.78	96.86 ± 22.30^*^ 45.81 – 145.34	97.89 ± 24.72^*^ 47.32 – 146.51
Male	49.64 ± 19.50^*^ 18.71 – 88.75	59.98 ± 16.74 33.01 – 91.39	63.02 ± 15.92 24.52 – 93.52	66.65 ± 12.72^*^ 48.42 – 86.49	58.33 ± 19.89 25.66 – 92.20
**Max.displacement (mm)**	4.45 ± 2.32^*^ .19 – 9.56	2.76 ± 1.66 .10 – 8.04	3.07 ± 1.90 .37 – 9.56	2.83 ± 1.65^*^ .73 – 7.17	2.82 ± 1.65^*^ .22 – 8.17
Female	3.93 ± 2.39^*^ .19 – 8.42	2.34 ± 1.42 .10 – 4.99	2.49 ± 1.47 .37 – 5.68	2.69 ± 1.73 73 – 7.17	2.46 ± 1.65^*^ .22 – 8.17
Male	5.10 ± 2.07^*^ 1.47 – 9.56	3.25 ± 1.81 .60 – 8.04	3.42 ± 2.07 .44 – 9.56	3.14 ± 1.44^*^ 1.04 – 5.95	3.57 ± 1.41^*^ .66 – 5.98
**Contraction time (ms)**	38.80 ± 12.16^*^ 13.91 – 84.55	39.14 ± 17.20 10.19 – 79.80	36.96 ± 14.57 13.32 – 74.68	46.82 ± 14.23^*^ 16.37 – 70.62	46.31 ± 14.59* 19.83 – 76.18
Female	39.40 ± 13.80 13.91 – 84.55	44.78 ± 18.74 10.19 – 79.80	41.72 ± 15.85 14.84 – 74.68	48.40 ± 13.28 20.52 – 70.62	46.78 ± 15.46 21.00 – 76.18
Male	38.05 ± 9.90 19.12 – 53.67	32.51 ± 12.60 15.51 – 62.32	34.04 ± 13.15 13.32 – 73.71	43.10 ± 16.17 16.37 – 69.00	45.31 ± 12.99 19.83 – 62.51
**Grip strength (kg)**	36.98 ± 10.03^*^ 18.00 – 66.13	39.61 ± 11.25 22.43 – 68.63	41.77 ± 13.20 17.67 – 78.73	32.26 ± 9.59^*^ 18.93 – 61.47	29.32 ± 9.37^*^ 12.77 – 57.10
Female	30.98 ± 6.56^*^ 18.00 – 48.20	31.10 ± 4.49 22.43 – 39.73	30.06 ± 6.41 17.67 – 44.13	26.99 ± 4.40^*^ 18.93 – 38.20	46.78 ± 15.46^*^ 12.77 – 33.13
Male	44.67 ± 8.34 25.30 – 66.13	46.61 ± 8.06 36.53 – 68.63	48.95 ± 10.95 29.40 – 78.73	43.45 ± 7.83 32.13 – 61.47	39.74 ± 8.88 28.77 – 57.10
**5 × chair rise (secs)**	5.86 ± 1.59^*^ 3.33 – 10.68	6.14 ± 1.58 3.29 – 11.51	6.29 ± 1.38 4.00 – 10.30	6.66 ± 1.51^*^ 4.00 – 10.80	7.42 ± 1.92^*^ 4.52 – 12.20
Female	5.69 ± 1.54^*^ 3.33 – 9.33	6.19 ± 1.20 4.15 – 8.53	6.26 ± 1.36 4.10 – 8.90	6.85 ± 1.72^*^ 4.00 – 10.80	7.52 ± 2.08^*^ 4.52 – 12.20
Male	6.07 ± 1.65 3.94 – 10.68	6.08 ± 1.96 3.29 – 11.51	6.31 ± 1.41 4.00 – 10.30	6.24 ± .85 4.60 – 8.30	7.20 ± 1.53 5.40 – 10.65
**1-min chair rise**	53.47 ± 13.58^*^ 28.00 – 78.00	49.30 ± 12.14 26.00 – 76.00	48.20 ± 11.74 27.00 – 80.00	45.00 ± 10.47^*^ 18.00 – 67.00	40.61 ± 11.64^*^ 20.0 – 70.00
Female	53.88 ± 13.91^*^ 28.00 – 78.00	47.52 ± 10.71 32.00 – 68.00	48.37 ± 11.54 31.00 – 75.00	42.29 ± 10.13^*^ 18.00 – 62.00	40.29 ± 11.40^*^ 20.00 – 70.00
Male	52.94 ± 13.34^*^ 32.00 – 78.00	51.39 ± 13.56 26.00 – 76.00	48.10 12.05 50.75 – 80.00	50.75 ± 8.99 35.00 – 67.00	41.31 ± 12.51^*^ 25.0 – 66.00
**METs**	4420.48 ± 2452.66^*^ 99 – 14,319	2884.12 ± 1776.39^*^ 446 – 9225	3232.47 ± 2187.42^*^ 0 – 9996	2660.67 ± 1937.10^*^ 132 – 10,890	2699.68 ± 1747.85^*^ 438 – 11,070
Female	4445.82 ± 2036.551^*^ 495 – 8244	2564.92 ± 1866.13^*^ 693 – 9225	2405.92 ± 1163.00^*^ 462 – 5772	2208.56 ± 1239.75^*^ 132 – 5772	2445.86 ± 1333.65^*^ 438 – 5346
Male	4388.02 ± 2935.65 99 – 14,319	3244.96 ± 1634.32 446 – 7812	3765.73 ± 2523.64 0 – 9996	3621.41 ± 2725.52 1512 – 10,890	3254.91 ± 2381.71 822 – 11,070

^*^statistically significant differences between the 18-29 yr age and the other age bands = *p* < 0.05

Values are presented as the mean ± standard deviation. The minimum and maximum values are presented underneath the mean ± standard deviation. The values for the whole sample are presented in the first row for each of the characteristics

### Analysis of muscle morphology, muscle function and functional capability between age categories

All indices of muscle morphology, muscle function and functional capability were significantly different between the youngest (18-29yrs) and both the 50–59 age band (p values: MT, PA, MQ and Dm = *p* < 0.001; FL = 0.006; Tc = 0.022; 5 × CR = 0.045; 1 min CR = 0.001) and the 60-70 yr age band (*p* < 0.001 for all variables).

An analysis of the female participants revealed no significant differences (*p* = 0.267) in the Tc between the youngest and oldest adults. However significant differences occurred between the youngest adults (18-29yrs) and oldest (60-70yrs) adults for every other variable measured (*P* values: MT, PA, FL, MQ, GS, 5 × CR and 1 min CR = *p* < 0.001; Dm = 0.023).

An analysis of the male participants revealed no significant differences were found between the age categories for the 5 timed chair rise test (*p* = 0.161), echo intensity (*p* = 0.659), Tc (*p* = 0.310) or GS (*p* = 0.367). Significant differences occurred between the youngest adults (18-29yrs) and oldest (60-70yrs) adults for the other variables measured (P values: MT = *p* < 0.001; PA = 0.016 FL = 0.007; Dm = 0.033; 1 min CR = 0.042).

### Relationships between age and muscle morphology, contractile properties, strength and functional capability

Age was associated with all dependent variables for the whole sample (*r*_s_ = 0.20- 0.56, *p* < 0.05) and females (*r*_s_ = 0.19 -0.71, *p* < 0.05), see Table 3. Tc and GS were not associated with age in the males (*p* > 0.05). The largest correlation coefficient was observed between age and MT for the whole sample, females and males. Overall, lower correlations were found in the males compared to the females, specifically, moderate correlations were only observed between age and MT (*r*_s_ = -0.40) and PA (*r*_s_ = -0.31) in the males, the remaining variables had small correlation coefficients (*r*_s_ = 0.10—0.27).

**Table 3:** Spearman’s rank correlations (r_*s*_) between age and each dependent variable

		**All**	**Female**	**Male**
MT	**r**	-.56^*^	-.71^*^	-.40^*^
PA	**r**	-.50^*^	-.62^*^	-.31^*^
FL	**r**	-.30^*^	-.29^*^	-.27^*^
MQ	**r**	.40^*^	.52^*^	.26^*^
Dm	**r**	-.24^*^	-.21^*^	-.27^*^
Tc	**r**	.20^*^	.19^*^	.13
GS	**r**	-.27^*^	-.43^*^	-.14
5 × CR	**r**	.30^*^	.35^*^	.22^*^
1 min CR	**r**	-.32^*^	-.38^*^	-.21^*^

^*^p < 0.05

*r*_s_ = Spearman’s rank correlation coefficient

*MT* muscle thickness, *PA* pennation angle, *FL* fascicle length, *MQ* muscle quality, *Dm* maximal displacement, *Tc* total contraction speed, *GS* grip strength, *5 X CR* 5 timed chair rise, *1 min CR* 1 min chair rise

## Analysis of variance

**Fig. 2:**
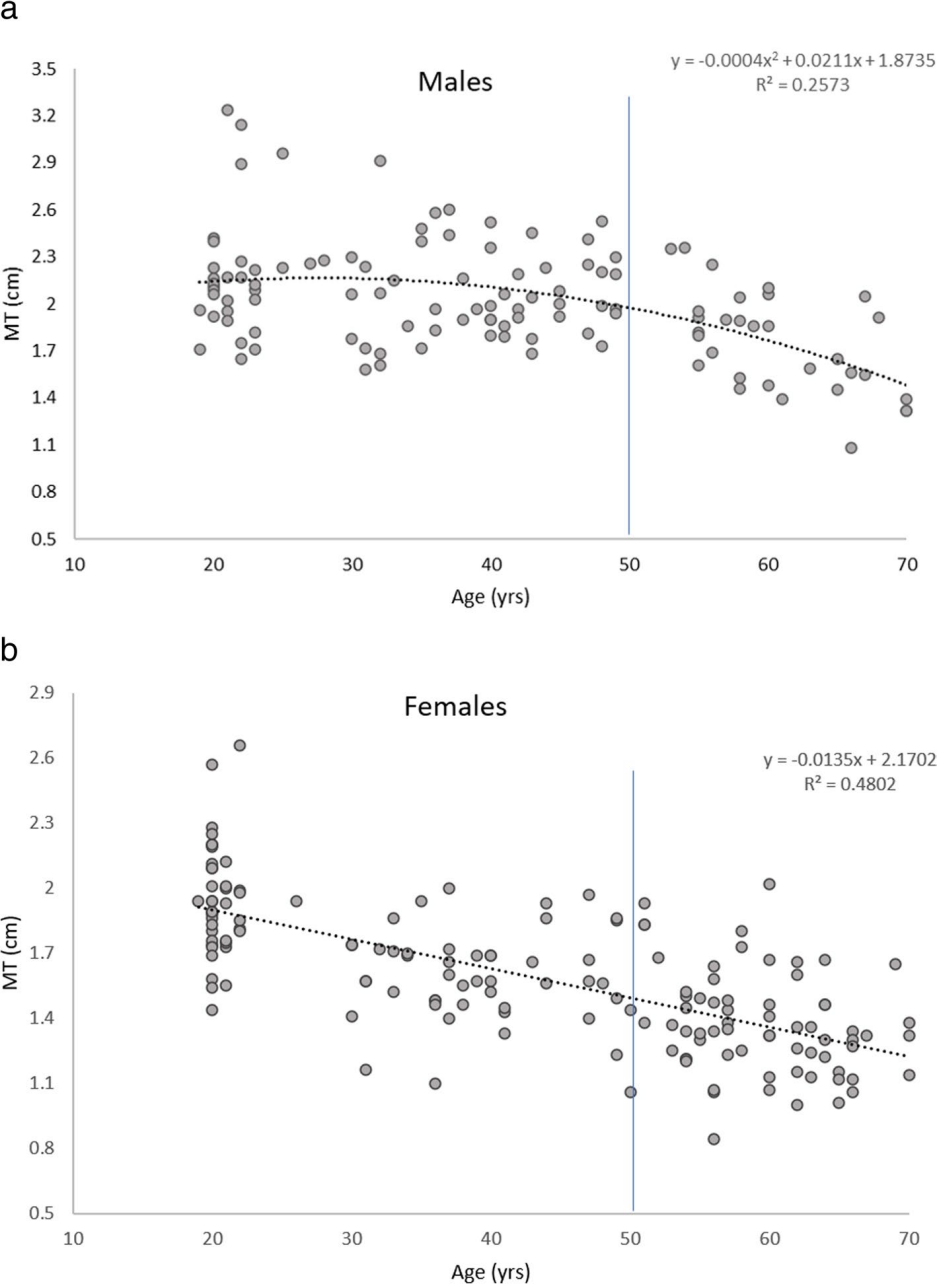
**a** and **b** Scatter plots to illustrate the relationship between age and MT in the males and females. The blue line represents the time point when differences in MT were noticeable from the youngest adults

**Fig. 3:**
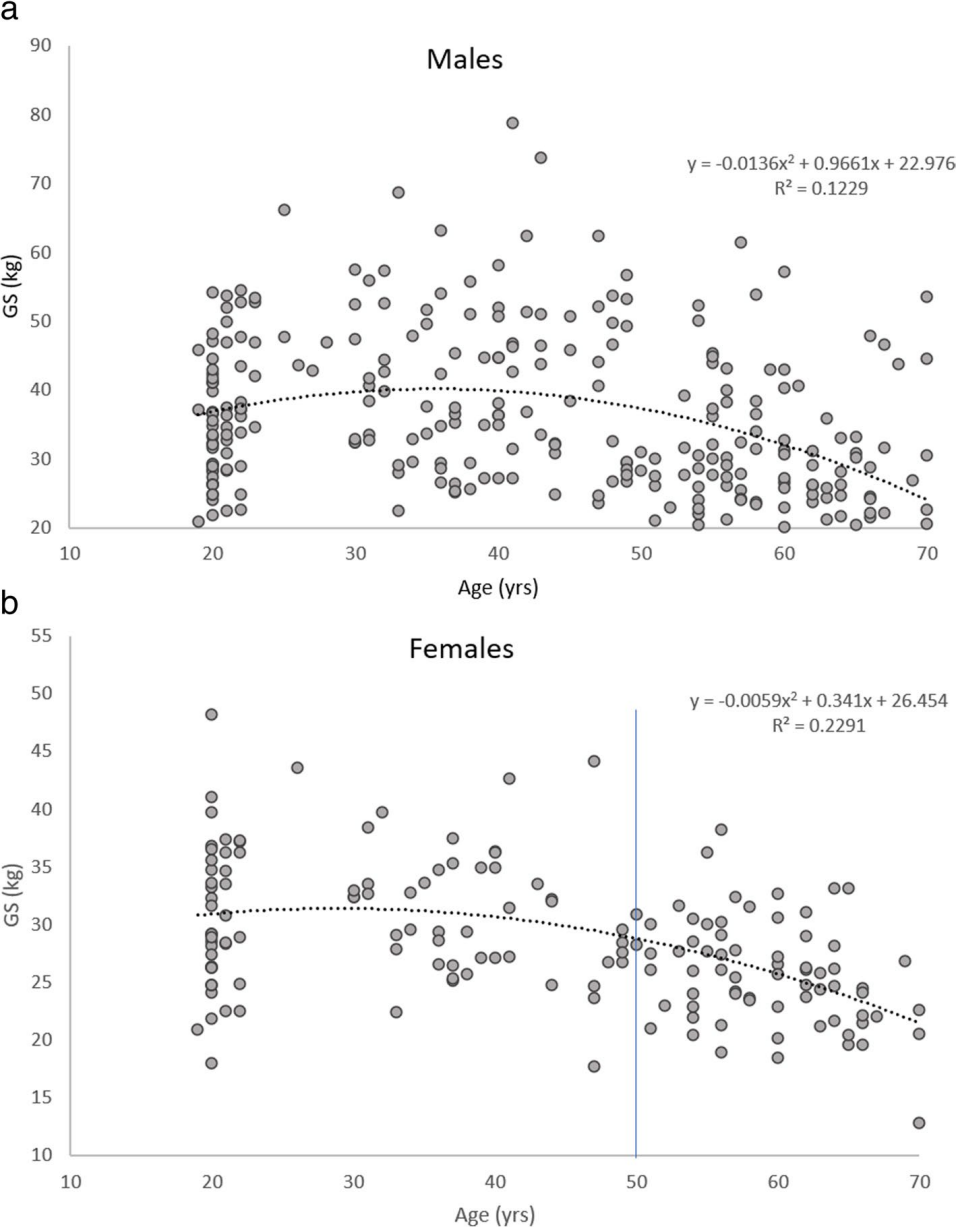
a and 3b Scatter plots to illustrate the relationship between age and GS in the males and females. The blue line represents the time point when differences in GS were noticeable from the youngest adults

Curvilinear relationships were observed between age and the following variables: MT (whole sample and males) and GS (whole sample, females and males). This was confirmed when age squared significantly contributed to the model for the aforementioned variables. Visual representations of the associations and regressions between age and MT and GS in the females and males can be seen in Figs. 2a, b, 3a and b.

Negative linear relationships were observed between age and the following variables: MT (females), PA (whole sample, females and males), FL (whole sample, females and males), Dm (whole sample, females and males) and the 1-min chair rise test (whole sample, females and males). Age did not statistically significantly account for any variances in the 5 timed chair rise test in the males (p > 0.05). Positive linear correlations were observed between age and MQ (whole sample and females), Tc (whole sample, females and males) and the 5 timed chair rise (whole sample and females).

The largest slope of regression line was observed between age and MT was in the females (r^2^ = 0.48, *p* < 0.05). The larger r^2^ value in the females (r = 0.48) compared to the males (r = 0.26) indicates that age accounted for a larger variance in MT in the females compared to the males. Please see S2 for the full regression analysis.

**Fig. 4:**
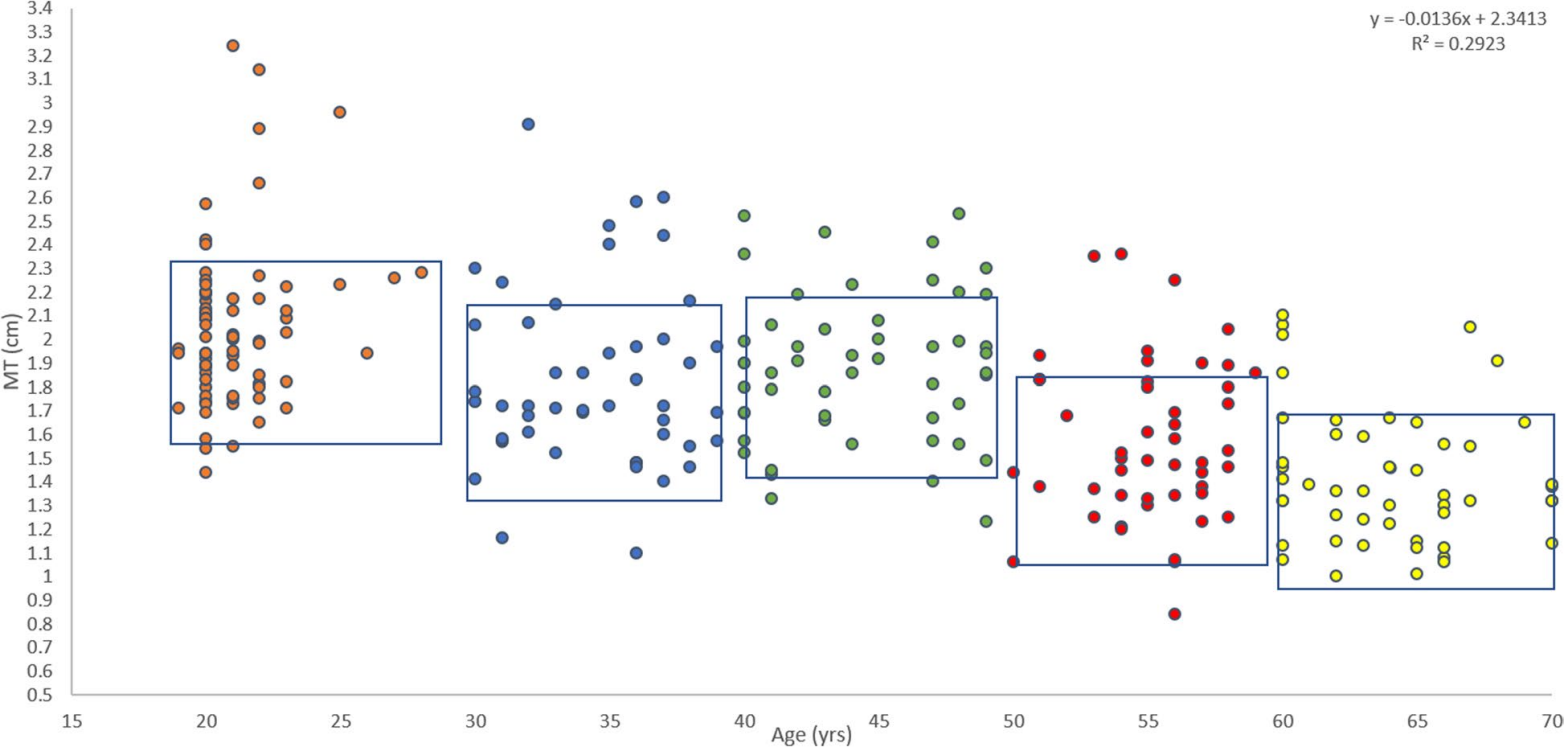
A scatter graph to illustrate the association between age and measurements of MT across 18-70yrs of age. The individual dots have been colour coded to represent the corresponding age band. The boxes represent the mean ± SD measurement of MT for each of the age bands

Figure 4 displays the association between age and MT for the whole sample across 18-70yrs. Visual inspection of Fig. 4 shows differences in MT across the age range. A difference in mean MT of 0.01 cm per annum is shown by the r equation.

### Multiple regression analysis of age and physical activity levels as predictors of changes in muscle morphology, function and functional capability

Table 4 presents the findings from the multiple regression analysis. It was revealed that when combined, age and physical activity were significant predictors of changes in all the variables. The r^2^ values demonstrate that the model (the interaction between age and physical activity) explained a larger variance in each of the dependent variables for the whole sample (r^2^ = 0.04—0.32), compared to either age (r^2^ = 0.04—0.30) or physical activity alone (r^2^ = 0.03—0.10).

**Table 4:** Multiple regression analysis summary for age and physical activity as predictors for muscle morphology, function and functional capability

	***B***	**SE *B***	**Β**	***T***	**r**^**2**^	**Adj. r**^**2**^
**MT**						
Age	-0.12	.00	-.49	-9.38^***^	.32^***^	.31^***^
METS	3.36	.00	.18	3.47^***^		
**PA**						
Age	-.06	.00	-.43	-7.83^***^	.25^***^	.24^***^
METS	.00	.00	.15	2.67^***^		
**FL**						
Age	-.02	.00	-.29	-4.85^***^	.11^***^	.10^***^
METS	3.99	.00	.09	1.42^***^		
**Echo intensity**						
Age	.55	.89	.34	6.18^***^	.24^***^	.23^***^
METS	-.00	.00	-.27	-4.77^***^		
**Dm**						
Age	-.03	.00	-.24	-3.87^***^	.10^***^	.09^***^
METS	.00	.00	.15	2.46^***^		
**Tc**						
Age	.18	.06	.20	3.13^***^	.04^***^	.03^***^
METS	.00	.00	-.02	-.27		
**GS**						
Age	-.13	.04	-.18	-3.04^***^	.10^***^	.09^***^
METS	.00	.00	.21	3.47^***^		
**5 timed chair rise**						
Age	.03	.00	.28	4.68^***^	.10^***^	.09^***^
METS	–7.54	.00	-.10	-1.63		
**1 min chair rise**						
Age	-.26	.05	-.32	-5.41^***^	.13^***^	.12^***^
METS	.00	.00	.09	1.50		

Abbreviations: *B* unstandardized beta, *SE B* standard error for unstandardized beta, *β* standardised beta, *t* t test statistic, ^***^ = *p* < .001

## Discussion

This is the first study to evaluate indices of VL muscle morphology, muscle function and lower extremity functional capability in a sample of healthy adults aged 18-70yrs (with ≥ 50 adults per age band). Age was negatively associated with vastus lateralis MT, PA, FL Dm, upper body strength and lower extremity functional capability. Also, there were positive associations between age and VL echo intensity and the total contraction time, yet the magnitude of the correlation coefficients between age and the indices of muscle morphology, function and functional capability were not the same. In particular, MT was the variable which demonstrated the largest association with age for the whole sample. Comparisons of age-related differences between the age bands revealed that it was not until the 50–59 age band when differences in all of the variables were statistically different to the youngest adults (18-29yrs). We suggest that the statistically significantly lower physical activity levels and lower number of weight-bearing exercise sessions may be a possible reason, alongside natural age-related changes, for the differences in those > 50yrs. Gender analysis revealed larger age-related differences and associations with age in the females compared to the males. However, males were more active compared to the females, suggesting that physical activity may have played an important role in preventing age-related differences in muscle health in the males.

Curvilinear relationships were observed between age and MT and age and GS in the whole sample and the males, suggesting that MT and GS do not peak in the third decade and then differ linearly across the subsequent age bands. This is consistent with previous research studies which also reported curvilinear declines in GS in both females and males [30–32]. Together, age and physical activity were contributing factors to differences observed in muscle morphology, contractile properties, strength and functional capability. Yet, age remained a significant correlate of the variables, more so than physical activity, suggesting that differences in muscle morphology, muscle function and lower extremity functional capability as a result of age are inevitable, both in females and males.

### Age was more strongly associated with MT compared to the other measures of muscle health

Findings from this study are suggestive that MT undergoes larger age-related differences compared to the other indices of muscle health. This is an important finding because MT underpins laboratory muscle function and functional capability [16]. Furthermore, research studies have suggested that alterations in muscle mass lead to subsequent impairments in the contractile elements of the muscles and muscle strength, and therefore physical performance [33].

Larger age-related differences in MT compared to GS were found between the youngest (18-29yrs) and oldest adults (60-70yrs). When bivariate correlations were conducted to control for the effect of height, weight and physical activity, the correlations were still stronger between age and MT compared to age and GS, suggesting that age accounts for larger differences in MT compared to GS. It could be argued that handheld dynamometry may not be the most appropriate tool to detect changes in muscle strength of healthy adults [34] as the lower limbs undergo larger age-related declines compared to the upper limbs [35]. Recently, the EWGSOP recommended the 30-s chair rise test as a proxy measure of lower limb muscle strength [10]. Healthy younger adults may not struggle to rise out of a chair continuously for 30 s, resulting in the test not being able to detect the degradation of functional capability in younger adults. It has been suggested that extended timed tests, e.g. a 900 m gait test or a 1-min chair rise test, may be more applicable, as it allows individuals to work to their maximum for a longer period of time, which may allow for the changes in functional capability to be identified [11]. Hence the 1-min chair rise test was performed in this study. Considering the 1-min chair rise test as a proxy measure of lower limb strength, larger age-related differences in mean MT between the youngest and oldest adults were found compared to lower extremity functional capability (1-min chair rise), at 29% and 24% respectively. These findings provide evidence that measurements of MT are important when investigating age-related differences in muscle health.

Interestingly, within this study, the rate of change per decade in mean MT and mean GS after 50yrs was the same (9%), indicating an accelerated loss of strength after 50yrs. This is in line with Samson et al. [36] and Vianna et al. [32] who both reported an accelerated loss of muscle strength after 55yrs. A variety of factors including age, hormones [36], changes in the size of the muscle (MT) and reduced physical activity levels are plausible explanations for these larger differences in strength after 50yrs. Based upon the findings from this study, it could be suggested that future interventions should explore the possibility of implementing approaches to prevent age-related differences in MT and GS prior to 50yrs because after this time point differences in these variables, in particular strength, accelerate.

### Differences in muscle thickness, pennation angle, fascicle length, muscle quality, contractile properties, strength and lower extremity functional capability were not noticeable from the youngest age band (18-29yrs) until the 50-59 yr age band

Differences in the indices of muscle morphology (MT, PA, FL and echo intensity), function (contractile properties and GS) and functional capability (5 timed chair rise and 1 min chair rise tests) were not statistically significantly different from the youngest adults until 50yrs. These findings are consistent with previous findings from studies which reported muscle quantity [37], strength [30–32] and functional capability noticeably decline after 50yrs [5].

A possible reason for the differences observed may be the physical activity levels of the 18-29 yr olds and 50-59 yr olds. The younger adults (18-29yrs) were more active than those aged 50-59yrs. Furthermore, a higher total number of weight bearing exercises such as strength training, high intensity interval classes, boxing and running were reported in the youngest (18-29yrs) adults compared to those aged 50-59yrs, 63 *vs.* 41. On the other hand, a higher total number of lower weight-bearing exercises, such as swimming, cycling, walking and yoga, were reported in those aged 50-59yrs (56) in comparison to the youngest (18-29yrs) adults [17]. The benefits of resistance training on the morphology and function of the muscle have been well documented [38–43]. From the above evidence, it seems reasonable to suggest the lower intensity exercises, including less weight bearing exercises, may have influenced the age-related differences in the variables of muscle health after 50yrs. Hence, both the frequency and type of physical activity plays an important role in mediating the age-related differences in muscle morphology, function and functional capability. One implication of these study findings is that interventions aimed at preventing age-related differences in muscle health may need to be implemented prior to 50yrs and that physical activity may need to play a part in these interventions.

### The interaction between age and physical activity account for a larger variance in the variables compared to age and physical activity alone

The inevitable effect of age on skeletal muscles is supported by research from Piasecki et al. [44], who provided evidence of natural age-related remodelling of motor units irrespective of physical activity levels. Although age accounted for a larger variance in the measures of muscle health within this study compared to physical activity, it is not known whether the age-related differences and associations observed would have been larger if the sample included within this study were not as physically active. Therefore, it could be suggested that whilst the effect of age on the skeletal muscle is unavoidable, physical activity plays a key role in mediating the declines in muscle health, not to mention the other benefits of physical activity on the reducing the chance of metabolic and cardiovascular diseases, musculoskeletal conditions, obesity and cognitive impairments [45].

### Gender comparisons

Larger associations and age-related differences in the indices of muscle morphology, function and functional capability were found in the females compared to the males. Females and males are different in many ways, including genetics, physical and physiological make up, for example males naturally have a larger amount of muscle mass compared to females [37]. Within this study, males were taller, heavier and had higher mean measures of muscle morphology (except for echo intensity values), strength and lower extremity functional capability relative to the females. When controlled for height, weight and physical activity levels, most of the correlations between age and the variables were still stronger in the females compared to the males. Thus, it could be suggested that females undergo larger age-related differences in muscle health compared to males. That said, the males were more highly active compared to of the females). Thus, the physical activity levels of the males may be a reason for the smaller age-related differences in the males compared to the females. The findings highlight that future studies investigating age-related differences and declines in muscle health should include both females and males, and that the findings between the genders cannot be generalised to one another.

### Limitations and future directions

Upper body strength was measured using handheld dynamometry in this study and a proxy measure of lower limb was measured using the 1-min chair rise test as proposed by the EWSOP [10]. Using measurements of upper body strength to reflect lower limb strength changes comes with its limitations, this is because research studies have provided evidence of the larger strength declines in the upper leg muscles, specifically the anterior thigh muscles, compared to the muscles of the upper arm [46]. This may be a possible reason for the smaller differences in GS compared to MT observed within this study.

This was a cross-sectional study design and allowed for age-related characteristics and differences in variables of muscle health to be determined in a sample of healthy adults. However, the specific decline in the measures of muscle health across 18-70yrs cannot be ascertained from this study. Therefore, whilst this study reported age-related differences across the various age bands, there is a need for a future study/studies to determine the age-related decline in measurements of muscle morphology, function and functional capability in healthy adults aged 18-70yrs.

## Conclusion

In conclusion, the present study provides preliminary evidence that age-related differences in the indices of muscle morphology, function and functional capability are not statistically different from the youngest adults until the 50–59 age band. The study found that age was associated with differences in indices of muscle morphology, function and functional capability. However, these variables do not differ similarly across the lifespan, in particular the evidence was suggestive that the thickness of the VL changed the most with age across the adult lifespan. From these initial findings, it could be suggested that interventions aimed at preventing sarcopenia and frailty should be targeted at those aged 50yrs. The findings also provide suggestive evidence that age-related differences in the indices of muscle morphology, function and functional capability cannot be generalised between genders. Although the effects of age on indices of muscle morphology, function and functional capability are inevitable, physical activity played an important role in mediating differences in the aspects of muscle health within this study. Therefore, these findings further support the use of physical activity as a non-medical intervention to reduce the risk of sarcopenia. The findings from this cross-sectional study provide insights which could be used to inform the design of future studies aiming to develop normative reference ranges for the aspects of muscle health across the adult lifespan, as well as studies aiming to devise interventions to prevent sarcopenia in females and males.

## Supplementary Information

**Additional file 1:** **Supplementary file S1. **Operationaldetail for the measurements of muscle morphology, function and functionalcapability. 

**Additional file 2:** **Supplementary file S2. **Furtherstatistical tests. 

## Data Availability

Underlying research materials related to our paper (for example data, samples or models) can be accessed by contacting i.jacob@leedsbeckett.ac.uk.
