# Supplementary material for: Age-related differences of vastus lateralis muscle morphology, contractile properties, upper body grip strength and lower extremity functional capability in healthy adults aged 18 to 70 years
Source: BMC Geriatr. 2022 Jun 29;22:538. doi: 10.1186/s12877-022-03183-4 (PMC9241209; doi:10.1186/s12877-022-03183-4)
Supplement: Supplementary file 1 — Additional file 1: Supplementary file S1. Operationaldetail for the measurements of muscle morphology, function and functionalcapability. [file 12877_2022_3183_MOESM1_ESM.docx]

**Supplementary file S1: Operational detail for the measurements of muscle morphology, function and functional capability.**

**Ultrasonography:**

The protocol published by Jacob et al (2020) was followed to prepare the participants, capture the images and measurement MT, PA, FL and echo intensity.

| Participant preparation | Protocol (technique) |  |
| --- | --- | --- |
| 1.Standardised measurement site using anatomical landmarks | 1. Locate two anatomical landmarks and mark with dermatological pen 2. Proximal point- greater trochanter 3. Distal point- Lateral condyle of femur 4. Measure the distance between 2.a.i and 2.a.ii along the sagittal plane 5. Mark 50% of the total distance using a dermatological pen 6. During no. 2 ensure that the mark is on the belly of the muscle by extending the leg and palpating for the bulk of the muscle. Adjust the mark if needed but stay in line with the original mark. | **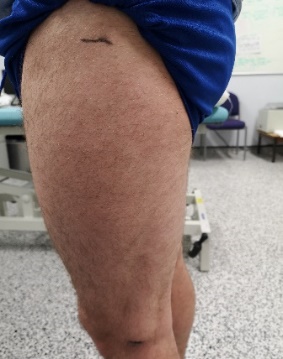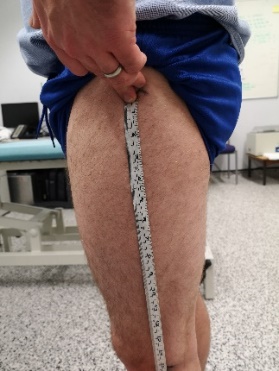** |
| 2.Patient position | 1. Supine 2. Knee extended 3. Ensure participant is fully relaxed | **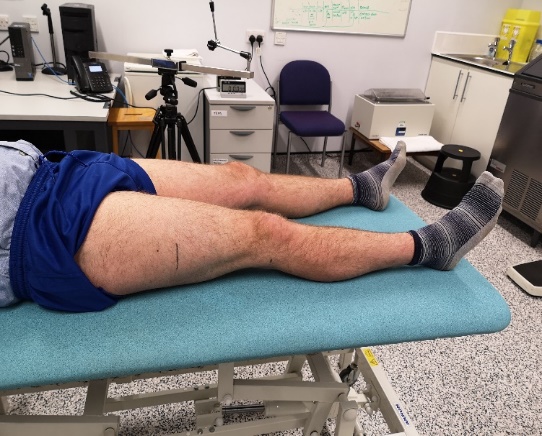** |
| Image capture | Protocol (techniques) |  |
| 3. Probe-skin interface | 1. Generous amount of ultrasound gel 2. Complete covering of probe head with a uniform amount of water-soluble ultrasound gel 3. Probe pressure 4. Uniform coverage of gel to reduce the compression of the skin and subsequently muscle thickness measurement 5. Place the probe head onto the location site, ensuring that the probe head is in contact with the skin without applying excessive pressure. | 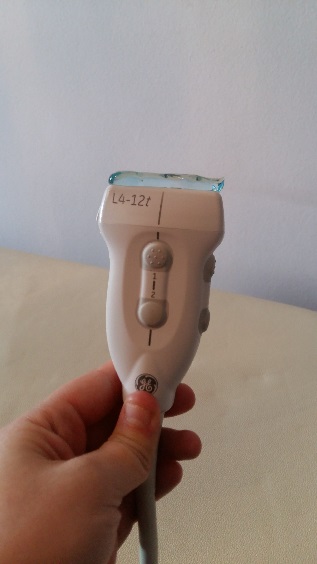  **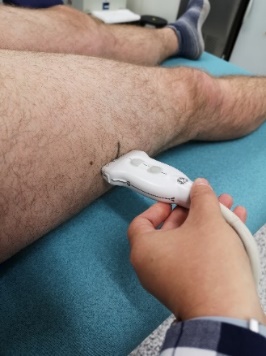** |
| 4.Probe orientation | 1. Probe orientated in the longitudinal axis of the thigh, parallel to the underlying tissue and femur bone in the sagittal plane, i.e. probe aligned to the fascicles 2. Ensuring that the arrow on the transducer is proximal to the greater trochanter. | **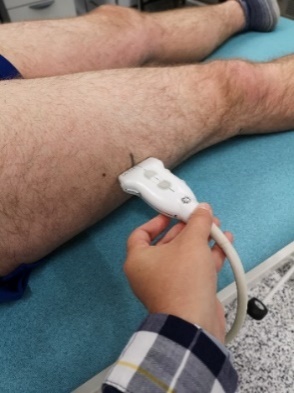** |
| 5. Identification of architectural landmarks | Muscle thickness, pennation angle & fascicle length:   1. A parallel superficial aponeurosis 2. A parallel deep aponeurosis 3. 3 clear fascicles extending from the deep aponeurosis to either the superficial aponeurosis or running off the edge of the screen. 4. Change the depth until these landmarks are identified. 5. Capture and save the image when all of these landmarks are identified. | **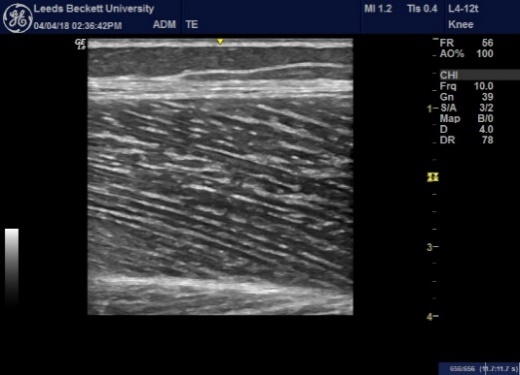**  ✓  **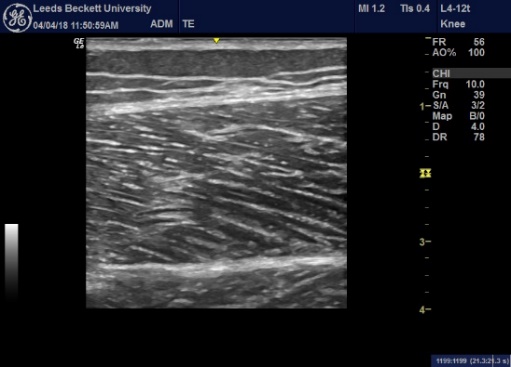**  🗶 |
| Measurement analysis | Protocol (technique) |  |
| 6. Muscle thickness measurement from captured image | On the image identify:   1. Superficial aponeurosis 2. Deep aponeurosis 3. Clear fascicles originating from the superficial or deep aponeurosis 4. Locate the middle point of the image (identifiable by an arrow on the top of the image) 5. Staying on the midpoint of the image, place the cursor on the inner aspect of 6.a.i. Drag the cursor in a straight line down towards the inner aspect of 6.a.ii. 6. Record the measurement in cm or mm. 7. Muscle thickness taken as the average of three consecutive measurements within 10%. 8. Repeat this for all images.   NB. Measurements of pennation angle, fascicle length and echo intensity can be taken from an image taken when the probe is orientated longitudinally.  Cross- sectional area, thickness and echo intensity measurements can be taken when the probe is orientated perpendicular to the muscle. | **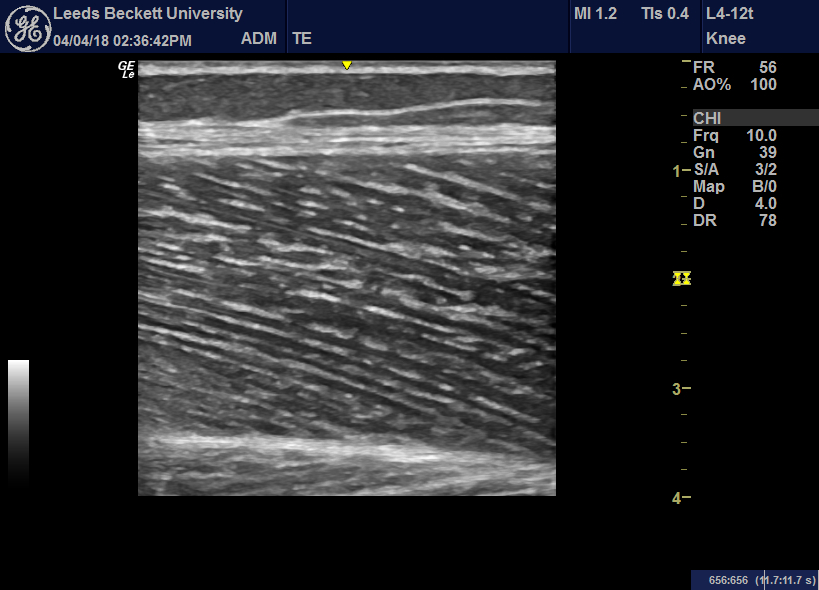**  c  e  d  b  a |

**Measurements of MT, PA and FL:**

The images were downloaded to imaging software (Image J, v.1.51k; National Institute of Health; Bethesda; USA) where one measurement of MT, PA, FL and MQ were taken. MT was determined as the perpendicular distance between the superficial and deep aponeurosis; PA was determined as the angle at which the fascicle inserted into the deep aponeurosis, and FL was determined as the length of the fascicular path from the superficial and deep aponeuroses. However, the entire length of the fascicles was not visible due to the fascicles extending off the images. FL was measured using the extrapolation method as shown in figure 1. The FL was measured as the length of the visible fascicle plus the estimated fascicle.


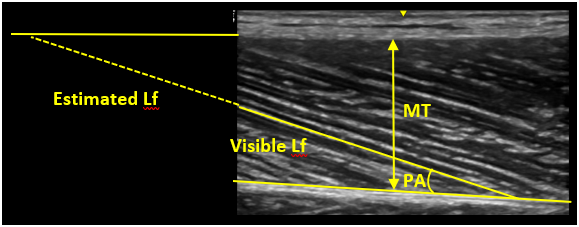


Figure 1 Longitudinal image of the vastus lateralis representing the measurement of muscle thickness (MT), pennation angle (PA) and fascicle length (Lf) via the linear extrapolation method

To measure the echo intensity Gray scale analysis was used. Image J was used to perform gray scale analysis. An area around the region of interest, the muscle belly, was drawn using the rectangular tool on Image J. Using the gray scale histogram analysis in Image J, a plot was constructed and the number of mean pixels within that region of interest was calculated. The mean pixels and standard deviation were recorded from a single image for each participant, see figure 2. The frequency gain and dynamic range settings were kept constant for every participant with only the depth changing.


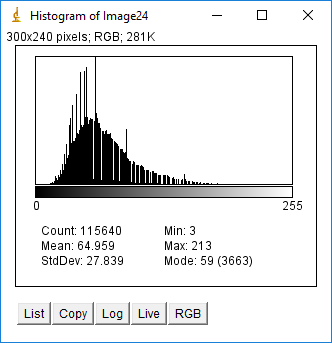


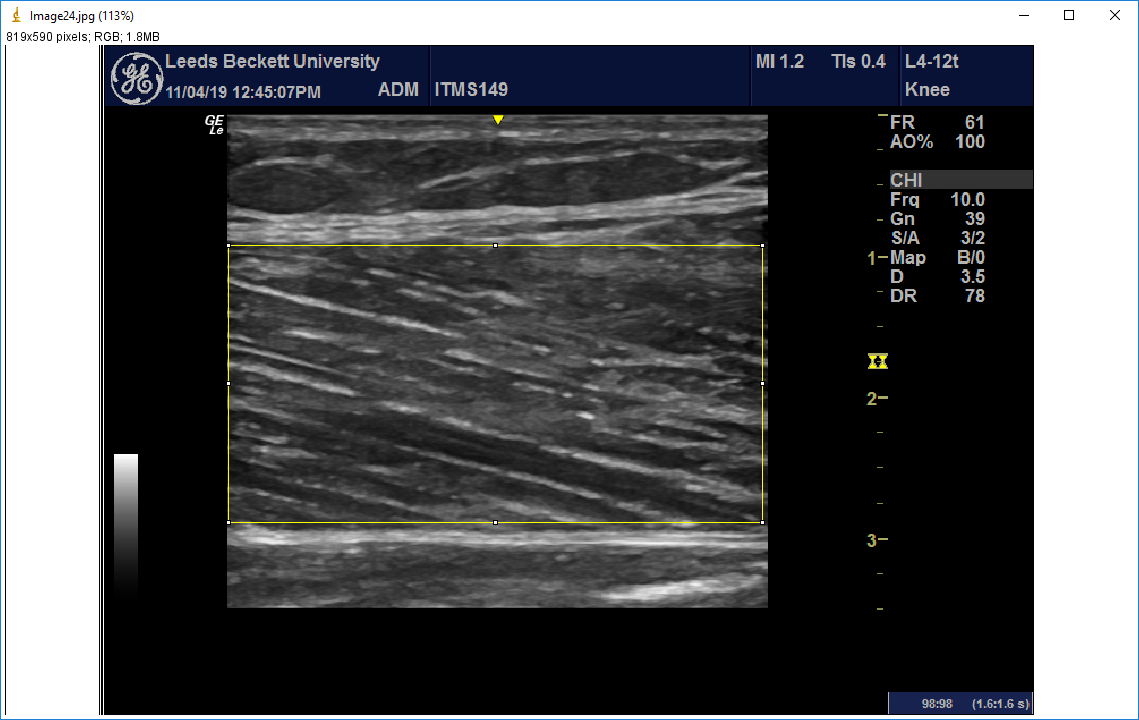


Figure 2- Longitudinal image of the vastus lateralis with the region of interest drawn around the muscle belly (yellow box). B- Histogram analysis of image A, including the mean pixels and standard deviation.

**TMG:**

Low amplitudes were used to familiarise the participant to the test. An amplitude of 30mA was followed by 35mA and 40mA; the familiarisation was terminated at this point. t. The spring bound probe was placed perpendicular to the mark on the muscle belly. The skin was deformed by the probe to~ 50% of the total length of the probe (determined by the naked eye).

Measurements were taken in a sequential order, starting at 30mA and increasing by 5mA every 10 seconds. On stimulation, a monophasic 1ms pulse stimuli is delivered to the muscle. After each stimulus, the investigator checked the curve on the graph shown on the laptop. The test was terminated if there was no further increase in maximal muscle displacement or if the maximum amplitude that the electrical stimulator was reached (100mA).

**Grip strength protocol:**

Set up: the hand dynamometer width will be altered to the participant’s hand.

Procedure:

1. The researcher will demonstrate the procedure to the participant before giving them the dynamometer.
2. The participant will be stood with the dynamometer held in the dominant hand.
3. The investigator will rest the bottom of the dynamometer in the palm of their hand to provide stability during the test.
4. Remind the subject that the handle will not move but to squeeze as hard as possible for 3-4 seconds.
5. Two sub maximal effort trials will be performed first with 15 second rest between each.
6. On the word GO the participant will be asked to squeeze with sub max or max effort, they will be asked to hold the force for 3-4 seconds. The investigator will encourage by saying “squeeze, squeeze”
7. After the sub max trials, they will perform 3 maximal effort trials, after each one a 30 second rest will be given.
8. Results with >5% variance will be excluded.

**Chair rise protocol:**

Set up: chair must be against a wall, with rubber feet to prevent it from slipping.

Procedure:

1. The researcher will demonstrate the test to the participant
2. The participant will sit in the middle of the chair with their feet easily touching the ground. The researcher will position the participant according to stature, someone who is small may not touch the backrest.
3. The feet should be shoulder width apart, they can be at an angle behind the knees with one foot in front of the other to help balance when standing.
4. The participant will have their arms crossed over the chest.
5. One chair rise is counted as: the participant rising from the chair knees extended at the top and then touching their bum back on the chair. As long as the bum touches the chair and the knees extend at the top this is accepted.
6. For the timed 5 times sit to stand the research will time how long it takes to perform the 5.
7. For the 1 minute repeated chair rise the researcher will count how many times they rise from the chair in a minute.

Any chair rises which were not executed properly were not counted. For both the tests, one full chair rise counted when their knees and hips extended fully from the seated position and their bottom touched the chair on the way back down.

*Key instructions:*

- “You are going to complete 2 tests, the first is how long does it take for you to rise out of a chair 5 times and the second is how many chair rises can you complete in a minute.2
- Remind them that it is a maximal test- how quickly can they perform 5 and how many they can perform in 1 minute.
- Demonstrate the technique - sit on the middle of the chair, feet planted on the ground, hands across the chest.
- One chair rise counts as- rising with the knees extended (standing tall) and then retunring with their bum touching the seat.
- “If you don’t extend your knees or touch the chair on the way down, this will not be counted.”
- “To start, I will count down from 3 and tell you when to go. I will count the 5 out loud and tell you when to stop. All you need to do is perform them as quickly as you can.”
- Count down from 3 and say GO.
- After they have completed the test, if they performed them wrong- repeat the test. If their technique was not perfect- remind them of what to do.
- “Ok, so for the next test you are going to see how many chair rises you can perform in 1 minute. I will count in my head, when you are half way I will tell you and when you have 10 seconds left.”
- The only encouragement you can give is “that’s it keep going”
- Do not say anything else or over motivate.
