# Supplementary material for: Age-related differences of vastus lateralis muscle morphology, contractile properties, upper body grip strength and lower extremity functional capability in healthy adults aged 18 to 70 years
Source: BMC Geriatr. 2022 Jun 29;22:538. doi: 10.1186/s12877-022-03183-4 (PMC9241209; doi:10.1186/s12877-022-03183-4)
Supplement: Supplementary file 2 — Additional file 2: Supplementary file S2. Furtherstatistical tests. [file 12877_2022_3183_MOESM2_ESM.docx]

**Supplementary file S2: Further statistical tests**

Table 1 presents the specific t test results for comparisons in mean MT, PA, FL, GS, the 5 timed chair rise and 1 minute chair rise between the 18-29yr age band and 60-70yr age band.

**Table 1: Differences in participant characteristics between the 18-29yr age band and 60-70yr age band. T test results.**

|  | **18-29yr age band mean ± SD** | **60-70yr age band mean ± SD** | **Δ between means** | **t**  **df = 122** |
| --- | --- | --- | --- | --- |
| **MT** | 2.05cm ± .34 | 1.42cm ± .28 | .63 | 10.87 * |
| **PA** | 15.28° ± 2.18 | 12.23° ± 1.79 | 3.05 | 2.18 * |
| **FL** | 8.34cm ± 1.02 | 7.33cm ± .89 | 1.01 | 5.62 * |
| **GS** | 36.98kg ± 10.02 | 29.32kg ± 9.37 | 7.66 | 4.3 * |
| **5 timed chair rise** | 5.86s ± 1.59 | 7.42s ± 1.92 | -1.56 | -4.95 * |
| **1 minute chair rise** | 53.47 ± 13.58 | 40.61 ± 11.64 | 12.86 | 5.5 * |
| Abbreviations  SD = standard deviation  Δ means= difference between the mean of the 18-29yr age band and the 60-70yr age band  t = t test statistic  df= degrees of freedom | | | | |

**Bivariate correlations**

Below are the bivariate correlations between age and the listed variables when height, weight and physical activity levels were controlled for.

- MT: whole sample r= -.58*; females r= -.71*; males r= -.45*.
- PA: whole sample r= -.56*; females r= -.61*; males r= -.32*.
- FL: whole sample r= -.26*; females r= -.18*; males r= -.32*.
- MQ: whole sample r= .30*; females r= .41*; males r= .19*.
- Dm: whole sample r= -.16*; females r= -.12; males r= -.20*.
- Tc: whole sample r= .22*; females r= .23*; males r= .26*
- GS: whole sample r= -.17; females r= -.32*, males r= -.16
- 5 x CR: whole sample r= .27*; females r= .32*; males r= .29*
- 1-minute CR: whole sample r= -.30; females r= -.38*; males r= -.27*

* p<0.05

**Regression analysis**

Table 2 presents the findings from conducting regression analysis between age and the list variables in the whole sample, females and males. The results show the curvilinear and linear relationships observed.

| **Table 2: Regression analysis between age and each of the variables** | | | |
| --- | --- | --- | --- |
|  | **Regression equation** | **R²** | **ANOVA** |
| **MT**  Whole  F  M | 2.044 + 0.003*age + 0.000*age²  2.170 - 0.014*age  1.873 + 0.021*age + 0.000*age² | .30  .48  .26 | F(2,272)= 58.66, p<.05  F(1,154)= 142.27, p<.05  F(2,114)= 19.42, p<.05 |
| **PA**  Whole  F  M | 16.579 – 0.065*age  16.205 – 0.072*age  16.607 – 0.043*age | .23  .37  .10 | F(1,273)= 78.16, p<.05  F(1,153)= 89.69, p<.05  F(1,115)= 12.72, p<.05 |
| **FL**  Whole  F  M | 8.758 – 0.020*age  8.495 – 0.018*age  8.757 – 0.02*age | .10  .10  .09 | F(1,273)= 29.79, p<.05  F(1,153)= 16.65, p<.05  F(2,114)= 8.58, p<.05 |
| **Echo intensity**  Whole  F  M | 45.876 + 0.668*age  44.102 + 0.703*age  44.404 + 0.691*age | .18  .18  .18 | F(1,273)= 56.76, p<.05  F(2,152)= 35.48, p<.05  F(2,114)= 6.26, p<.05 |
| **Dm**  Whole  F  M | 7.068 – 0.164*age  4.124 – 0.029*age  9.183 – 0.380*age | .10  .06  .08 | F(1,267)= 21.80, p<.05  F(1,151)= 10.13, p<.05  F(1,114)= 10.19, p<.05 |
| **Tc**  Whole  F  M | 33.560 + 0.187*age  37.095 + 0.168*age  58.685 + 1.700*age | .04  .03  .04 | F(1,266)= 11.19, p<.05  F(1,151)= 5.06, p<.05  F(1,113)= 4.50, p<.05 |
| **GS**  Whole  F  M | 22.620 + 0.979*age – 0.0148*age²  26.456 + 3.41*age – 0.006*age²  28.478 + 1.069*age – 0.014*age² | .12  .23  .12 | F(2,271)= 18.70, p<.05  F(2,152)= 22.74, p<.05  F(2,113)= 7.78, p<.05 |
| **5 timed chair rise**  Whole  F  M | 5.067 + 0.032*age  4.753 + 0.041*age | .10  .15  .03 | F(1,273)= 28.51, p<.05  F(1,154)= 26.89, p<.05  F(1,115)= 3.59, p>.05 |
| **1 minute chair rise**  Whole  F  M | 59.378 – 0.276*age  59.836 – 0.309*age  57.918 – 0.208*age | .12  .16  .06 | F(1,273)= 36.80, p<.05  F(1,154)= 29.99, p<.05  F(1,115)= 7.52, p<.05 |
